# Optimizing a Just-in-Time Adaptive Intervention to Improve Dietary Adherence in Behavioral Obesity Treatment: Protocol for a Microrandomized Trial

**DOI:** 10.2196/33568

**Published:** 2021-12-06

**Authors:** Stephanie P Goldstein, Fengqing Zhang, Predrag Klasnja, Adam Hoover, Rena R Wing, John Graham Thomas

**Affiliations:** 1 Weight Control and Diabetes Research Center The Miriam Hospital Providence, RI United States; 2 Department of Psychiatry and Human Behavior Alpert Medical School of Brown University Providence, RI United States; 3 Department of Psychology Drexel University Philadelphia, PA United States; 4 School of Information University of Michigan Ann Arbor, MI United States; 5 Holcombe Department of Electrical and Computer Engineering Clemson University Clemson, SC United States

**Keywords:** obesity, weight loss, dietary adherence, just-in-time adaptive intervention, microrandomized trial, mobile phone

## Abstract

**Background:**

Behavioral obesity treatment (BOT) is a gold standard approach to weight loss and reduces the risk of cardiovascular disease. However, frequent lapses from the recommended diet stymie weight loss and prevent individuals from actualizing the health benefits of BOT. There is a need for innovative treatment solutions to improve adherence to the prescribed diet in BOT.

**Objective:**

The aim of this study is to optimize a smartphone-based just-in-time adaptive intervention (JITAI) that uses daily surveys to assess triggers for dietary lapses and deliver interventions when the risk of lapse is high. A microrandomized trial design will evaluate the efficacy of any interventions (ie, theory-driven or a generic alert to risk) on the proximal outcome of lapses during BOT, compare the effects of theory-driven interventions with generic risk alerts on the proximal outcome of lapse, and examine contextual moderators of interventions.

**Methods:**

Adults with overweight or obesity and cardiovascular disease risk (n=159) will participate in a 6-month web-based BOT while using the JITAI to prevent dietary lapses. Each time the JITAI detects elevated lapse risk, the participant will be randomized to no intervention, a generic risk alert, or 1 of 4 theory-driven interventions (ie, enhanced education, building self-efficacy, fostering motivation, and improving self-regulation). The primary outcome will be the occurrence of lapse in the 2.5 hours following randomization. Contextual moderators of intervention efficacy will also be explored (eg, location and time of day). The data will inform an optimized JITAI that selects the theory-driven approach most likely to prevent lapses in a given moment.

**Results:**

The recruitment for the microrandomized trial began on April 19, 2021, and is ongoing.

**Conclusions:**

This study will optimize a JITAI for dietary lapses so that it empirically tailors the provision of evidence-based intervention to the individual and context. The finalized JITAI will be evaluated for efficacy in a future randomized controlled trial of distal health outcomes (eg, weight loss).

**Trial Registration:**

ClinicalTrials.gov NCT04784585; http://clinicaltrials.gov/ct2/show/NCT04784585

**International Registered Report Identifier (IRRID):**

DERR1-10.2196/33568

## Introduction

### Background

Behavioral obesity treatment (BOT), a first-line intervention for overweight and obesity, typically produces a 5% to 10% reduction in initial body weight [1,2]. However, many individuals lose less weight than expected, thereby negating the potential health benefits of weight loss (eg, reduced cardiovascular disease [CVD] risk and severity) [3,4]. These suboptimal outcomes can be, in part, attributed to nonadherence to the prescribed calorie goal and recommended dietary guidelines to reduce energy intake [5]. Research has shown that dietary lapses (ie, specific instances of nonadherence to BOT dietary goals) occur 3-4 times per week in BOT and are associated with poorer weight losses on average [6,7]. Although the ability to cope with temptation and prevent lapse has been associated with BOT success [8-10], there is insufficient evidence on how to provide these necessary skills for individuals to reduce dietary lapses in BOT.

Extant strategies to improve adherence in BOT (eg, stimulus control) require vigilance for potential lapse triggers and the ability to implement an effective plan to avoid lapse. Alternatively, just-in-time adaptive interventions (JITAIs) can proactively monitor lapse risk and provide support to prevent lapses in an adaptive manner and in exact moments of need [11,12]. Our team developed a smartphone-based JITAI that uses ecological momentary assessment (EMA) [13] to monitor triggers for lapses via repeated surveys throughout the day [14]. The JITAI analyzes EMA responses in real time using a machine learning algorithm to calculate the ongoing level of risk for lapsing and then delivers preventive intervention as needed. This JITAI has demonstrated feasibility and acceptability in two 8- to 10-week pilot studies [15,16]. Using simple intervention messages (ie, 1-2 screens of text), the JITAI was associated with *average* reductions in dietary lapses. However, the JITAI has not been evaluated for efficacy directly in the moments of heightened lapse risk, and there is little evidence (or theory) available to guide *which* interventions should be delivered in these moments of vulnerability to achieve maximum clinical benefit.

### Objectives

To develop a scientifically rigorous and maximally effective JITAI for dietary adherence, research must experimentally evaluate the proximal efficacy of theory-driven interventions for reducing lapses [17]. This paper describes the design of a microrandomized trial (MRT) to optimize a JITAI for dietary lapses by empirically determining which theory-driven interventions are effective in preventing lapses and contexts that could influence intervention effectiveness [18,19]. Each time a participant is determined to be at high risk for lapsing based on the JITAI’s algorithm, they will be randomized to either no intervention, a generic alert to risk, or 1 of 4 theory-driven intervention options to provide education on dietary goals, increase self-efficacy, enhance motivation, or improve self-regulation. Each participant can be randomized over 100 times during the study (based on the rate of algorithm-determined lapse predictions), which will efficiently provide the critical information required to optimize the JITAI [17,18]. The results of this study will inform an improved JITAI for lapses that can be evaluated in a future randomized controlled trial (RCT) and contribute to the broader evidence base of developing JITAIs for problematic eating behaviors (ie, understanding the relative efficacy of theory-based approaches to modifying behavior and informing dynamic theoretical models of behavior).

## Methods

### Study Aims and Design

This study aims to optimize a smartphone-based JITAI for dietary lapses by evaluating the efficacy of 4 theory-driven interventions on the proximal, immediate outcome of lapse during BOT. This study uses an MRT design because it is the most efficient experimental design to determine *which* interventions are efficacious *at a given moment in time* [18]. The use of the MRT, with more than 100 randomizations and observations of the outcome per participant, allows for the evaluation of each intervention condition with full statistical power [17]. In stage 1, the JITAI and MRT study procedures (including the microrandomization algorithm) will be tested with a small number of participants for 3 months to ensure proper functioning before proceeding to stage 2. In stage 2, adults with overweight or obesity and ≥1 CVD risk factor (eg, diagnosis of hypertension, hypercholesterolemia, and type 2 diabetes) will participate in a well-established 3-month web-based BOT (BOT + JITAI) with 3 months of JITAI-only follow-up. During BOT and follow-up, participants will use the smartphone-based JITAI consisting of the following: (1) EMA to assess lapses and relevant behavioral, psychological, and environmental triggers; (2) a machine learning algorithm that uses information gathered via EMA to determine real-time lapse risk; and (3) randomized administration of intervention to counter lapse risk. When an individual is at risk for lapsing, they will be randomized to no intervention, a generic risk alert, or 1 of 4 theory-driven interventions with skills training. The primary proximal outcome of interest will be the occurrence (or lack thereof) of dietary lapse, as measured by EMA [7], in the 2.5 hours following randomization. The secondary proximal outcomes of interest will be the passive measurement of eating characteristics (ie, duration, rate of eating, and count of bites taken in the 2.5 hours following randomization) via wrist-based monitoring for the first 14 days of treatment and subsequent 14-day periods at 3 and 6 months [20]. Contextual moderators will be explored to determine the circumstances under which interventions are more or less effective (ie, location, time of day, whether in active BOT or follow-up, and type of lapse triggers endorsed). JITAI engagement, satisfaction, and weight will be measured at baseline, 3 months, and 6 months. When the MRT is complete, stage 3 will consist of using the data to inform an optimized JITAI that selects the theory-driven approach most likely to counter lapse risk in a given moment. This study has 4 aims:

- Aim 1: evaluate the effects of any intervention (ie, theory-driven or generic risk alert) versus no intervention on the occurrence of dietary lapse in *each moment* when the lapse risk is predicted to be high.
- Aim 2: compare the effects of theory-driven interventions and generic risk alerts on the occurrence of dietary lapse.
- Aim 3: use the data from the MRT to optimize an algorithm for intervention delivery that will drive the JITAI in a future RCT examining the effects on overall weight change in an obesity treatment program.
- Exploratory aim: examine contextual moderators (eg, time, location, and active BOT or follow-up) of interventions.

### JITAI for Dietary Lapses

#### Overview

**Figure 1:**
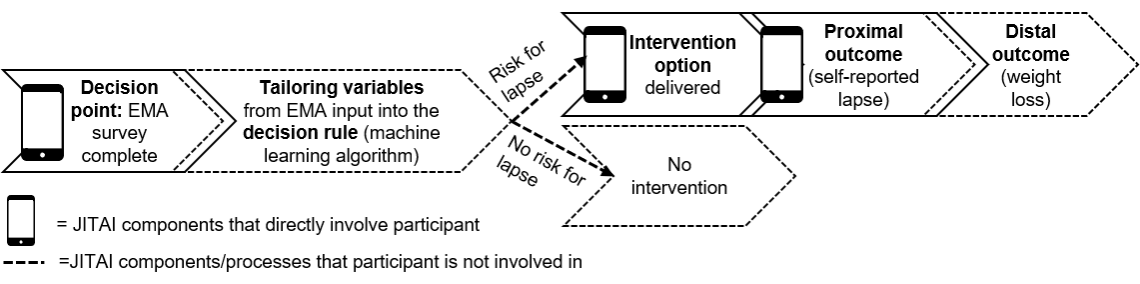
Conceptual model of just-in-time adaptive intervention components. EMA: ecological momentary assessment; JITAI: just-in-time adaptive intervention.

The JITAI for dietary lapses to be optimized by the current trial was developed in line with the conceptual framework set forth by Nahum-Shani et al [21]. According to their established framework, JITAIs should include the following components: decision points (times at which an intervention decision is made), tailoring variables (information that is used at a decision point to decide when and how to intervene), decision rules (algorithms deciding which intervention option to offer and for whom and when), intervention options, proximal outcomes (behaviors directly targeted by the JITAI), and distal outcomes (health conditions that are expected to improve as a result of targeting proximal outcomes). A conceptual model of the JITAI components in the current trial and how they work together to provide real-time adaptive intervention to prevent dietary lapses is shown in Figure 1.

#### Decision Points

As shown in Figure 1, the decision points in this trial occur immediately following the completion of each EMA survey [21]. EMA is well suited to inform decision points because the measurements of tailoring variables can be repeated over time in the changing context of everyday life, thus informing multiple opportunities for assessment and intervention [22]. Consistent with previous studies, the JITAI for lapses will prompt participants via vibration and audible tone to complete 6 EMA surveys throughout the day (anchor times at 8:30 AM, 11:00 AM, 1:30 PM, 4:00 PM, 6:30 PM, and 9:00 PM) [7,15,16]. Participants are given 90 minutes to respond to an EMA survey before it expires. The 6 EMA surveys inform 6 decision points each day for which an intervention *could be provided*. Randomization to an intervention option will only be triggered at a subset of decision points in which an EMA survey is completed, *and* lapse risk is judged to be elevated, which previous work has shown occurs approximately once per day on average [15].

#### Tailoring Variables

A JITAI tailoring variable is participant information that is used to decide (1) when to intervene (ie, help define the decision point) and (2) how to intervene (ie, which type of intervention to administer) [21]. The tailoring variables used to determine *when* to intervene in the proposed JITAI will be measured via 17 EMA survey questions that assess behavioral, psychosocial, and environmental triggers for lapse. Pilot studies confirmed that these 17 variables are feasible to assess via EMA and are suitable for predicting lapse in the JITAI (see the *Measures* section for a complete list of tailoring variables) [7,15,23]. The exploratory aim of this research is to identify other tailoring variables (eg, contextual moderators) to refine the JITAI by explaining *how* to intervene under specific risk conditions.

#### Decision Rule

The decision rule uses tailoring variables to identify the current state of vulnerability and specifies when it is appropriate to offer intervention [21]. Owing to substantial individual variability in what tailoring variables and at which thresholds indicate a heightened state of lapse risk, a machine learning algorithm informs the decision rule in this JITAI for lapses [6,24-26]. In formative work to develop this JITAI, a supervised machine learning approach was used to train an algorithm using previously collected data on tailoring variables and dietary lapses. Preliminary research revealed that ensemble classifiers, a series of C.5 decision tree algorithms, predicted the likelihood of reporting a lapse in the next EMA survey (in approximately 2.5 hours) with 72% specificity and 70% sensitivity [7]. This study also showed that combining group- and participant-level data is the most efficient approach to lapse prediction; therefore, the decision rule algorithm allows the JITAI to start with a base algorithm comprised of data from previous trials and then continuously adapt itself to the individual via incoming information. When piloting this JITAI for lapses, the decision rule algorithm predicted lapses with 80% negative predictive value (n=43) [15] and 76.5% accuracy (n=116) [16], thus indicating that it is ready for use in the current trial. At each decision point, the tailoring variables from a participant’s EMA survey will be uploaded to the JITAI platform (operating via PiLR Health, a product of MEI Research Ltd to execute EMA studies), which will process the data using the above-described decision rule algorithm (Figure 1). On the basis of these data, the algorithm will then predict whether or not a participant is likely to lapse in the following 2.5 hours. If the prediction for lapse is *yes*, then the participant will be randomized to 1 of 6 intervention conditions (ie, 4 theory-driven interventions, generic risk alert, and no intervention). If the prediction for lapse is *no*, then nothing will be done at that time because the participant is not in a state of heightened lapse risk.

#### Intervention Options

##### Overview

The intervention options in a JITAI should be driven theoretically or empirically [21]. Previous pilot studies to develop this JITAI for lapses have used simple—interventions (ie, 1-2 screens of text) based on a multitude of behavior change taxonomies. This study will improve upon previous work by using an a priori selected conceptual model of adherence behavior to guide theory-driven intervention options that are designed to be interactive and engaging.

**Figure 2:**
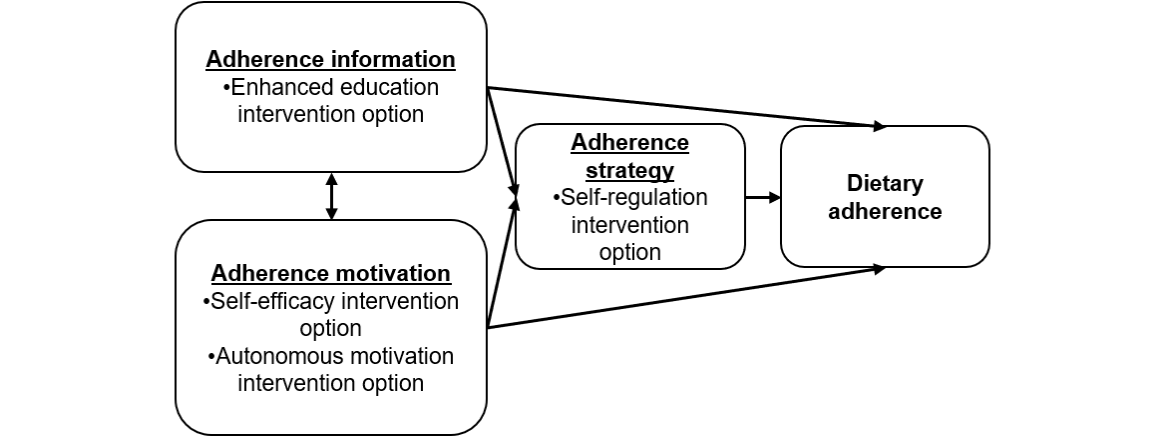
The information-motivation-strategy model that informed the just-in-time adaptive intervention options.

The theory-driven intervention options to be evaluated in this study were developed using the Information-Motivation-Strategy (IMS) model as a theoretical basis [27]. The IMS model extends and is grounded in several health behavior models (eg, Health Belief Model and Theory of Planned Behavior) and has been shown to be a valid approach to understanding adherence behaviors via meta-analytic reviews and large-scale trials [27,28]. The IMS model posits that there are 3 influences on adherence to health recommendations or guidelines: (1) information (ie, providing education on factors that influence adherence and treatment goals), (2) motivation (ie, motivating patients to carry out recommendations via self-efficacy and aligning to a person’s values), and (3) strategy (ie, strategizing with participants to ensure capability and ability to adhere). In addition to being an empirically validated model for studying adherence behavior, generally, the IMS model can flexibly incorporate theory-driven interventions with empirical support for dietary adherence, specifically. The IMS model also encourages tailoring within the categories, making it consistent with the JITAI framework. Figure 2 illustrates how the IMS model informed the following theory-driven intervention options to be tested within this MRT, focusing on enhanced education, self-efficacy, autonomous motivation, and self-regulation.

The selected intervention options act as companions to the web-based BOT program. They are designed to remind participants to use skills that they have already been taught through the web-based BOT (ie, self-monitoring, setting dietary goals, basic self-regulation skills, and problem-solving) or provide easy-to-digest new strategies to facilitate engagement in behavioral skills. Each intervention option (as well as the active comparator and generic alerts to risk) comprises a library of brief intervention modules that can be administered in any order. The variation in intervention content is expected to facilitate long-term engagement via reduced repetition and encourage well-rounded skill development [29,30]. If a participant is randomized to a theory-driven intervention option or active comparator condition, a module will be randomly selected from the library of that condition. Each module is designed to have the highest impact while minimizing burden. The content of these modules, which is hosted in the PiLR Health platform, consists primarily of 3- to 5-minute instructional videos that are interactive where possible (eg, prompting participant responses and using branching logic to tailor content) [12]. A description of the modules included in each theory-driven intervention option as well as active and inactive comparators is presented in subsequent sections.

##### Enhanced Education Intervention Option

The IMS model highlights the importance of participant knowledge in determining adherence [27,31]. Providing education on the link between dietary recommendations and health has improved dietary adherence among participants with CVD risk [32,33]. In particular, asking participants to repeat key points has been shown to increase the understanding of disease and enhance adherence to dietary guidelines [34,35]. A library of 6 independent modules was created; all modules seek to promote the understanding of health behavior links, improve health literacy by using brief quizzes, and remind participants of important elements of the BOT dietary goals. When participants are randomized to the enhanced education intervention option, they will randomly receive one of the following independent modules focused on (1) the role of dietary fat in health and strategies to reduce the consumption of saturated and trans fats, (2) the role of sodium in health and recommendations for sodium intake to reduce CVD risk, (3) the role of added sugars in health and strategies to reduce the consumption of foods with added sugar, (4) the role of small lapses in contributing to higher caloric intake for the day and strategies for reducing small lapses, (5) preventing lapse during a high-risk time by choosing foods that are low in calories and filling, *or* (6) the role of evening calorie consumption on health and weight loss (delivered in evenings only). This intervention option is expected to improve dietary adherence by reminding participants of important health goals and improving knowledge retention.

##### Self-efficacy Intervention Option

According to the IMS model, participants’ confidence in their ability to change behavior (ie, self-efficacy) is essential for adherence [27]. Self-efficacy has been robustly associated with improved weight loss and adherence to dietary recommendations, which provides strong justification for targeting it in the JITAI for lapses [36,37]. A library of 4 independent modules was created based on self-efficacy–based BOT [38], a multi-component intervention that has been shown to improve self-efficacy for weight loss via goal-setting, problem-solving, self-reward, and coping with difficult thoughts. When participants are randomized to the self-efficacy intervention option, they will randomly receive *one* of the following modules developed to emulate self-efficacy–based BOT: (1) setting attainable goals related to dietary adherence; (2) barrier identification for adhering to dietary goals along with a brief problem-solving exercise; (3) devising a small, non–food-related self-reward for adhering to the day’s dietary goal; *or* (4) a self-assessment of negative thoughts that could interfere with dietary adherence in the next several hours, along with suggested coping statements. This intervention option is expected to facilitate dietary adherence by improving self-efficacy in moments of heightened lapse risk, which will enhance motivation and the ability to engage in adherence strategies.

##### Autonomous Motivation Intervention Option

Another central tenant of the IMS model is that beliefs about the value of engaging in a behavior are critical to adherence [39-41]. A library of 4 independent modules was created based on principles from motivational interviewing (MI) and acceptance and commitment therapy (ACT) [42,43]. Both MI and ACT are widely used strategies for improving and maintaining motivation for health behavior change [44,45]. MI and ACT use a collaborative and nonjudgmental approach to identify valued directions and make mindful decisions about engaging in behaviors that are consistent with short- and long-term goals. When participants are randomized to the autonomous motivation intervention option, they will randomly receive *one* of the following modules: (1) guidance in identifying values related to healthy eating and weight control (eg, longevity and quality of life) and connecting those values to daily dietary goals, (2) exploring the short- and long-term consequences of choosing to lapse or stay the course (eg, “Take a moment to consider the effect on your longevity if you let your preferences for sweets determine your behavior”), (3) clarifying values and thinking about every behavior (including a lapse) as an *up* or *down* vote for their values, *or* (4) engaging participants in a brief self-assessment of motivation for dietary adherence. This intervention option is expected to facilitate dietary adherence by increasing the salience of participants’ beliefs about the importance of their dietary goals.

##### Self-regulation Intervention Option

The IMS model indicates that participants must have *capacity* and *ability* in order for adherence to occur [27]. The *capacity* to adhere, in particular to self-regulate dietary intake and thus prevent lapse, largely depends on the ability to maintain awareness of eating behavior [46,47]. The self-regulation approach to BOT has been extensively tested and encourages the self-regulation of dietary intake via increased prompts to intensify self-monitoring of dietary intake [48-50]. A library of 5 independent modules was developed to prompt self-monitoring and improve self-awareness. When participants are randomized to the self-regulation intervention option, they will randomly receive *one* of the following modules: (1) a prompt to self-monitor any foods before they are consumed in the next 2 to 3 hours, (2) introduce the *traffic light* model to improve the awareness of intake using a quick rule-of-thumb system especially when the risk for lapse is high [51], (3) increase the awareness of portion sizes during heightened lapse risk (eg, reading labels, weighing and measuring, and portion size guide), (4) provide a tutorial on noticing hunger and satiety cues and slowing down the rate of eating, *or* (5) prompt an awareness of end-of-day grazing or mindless eating that may lead to lapses (delivered during evenings only). The self-regulation intervention option is expected to facilitate the necessary self-regulation strategies required for dietary adherence.

##### Generic Risk Alert (Active Comparator)

A generic risk alert intervention option is included as an active comparator to the theory-driven intervention options, as it controls for the potential influence of receiving any notification of risk. For example, the notification alone could be expected to influence lapse risk via heightened awareness of behavior. A library of 3 generic risk alert messages was created (eg, “We have detected that your risk of lapsing from your weight loss diet is higher than usual and may require attention”). When participants are randomized to the generic alert active comparator, they will randomly receive *one* of these simple text-based messages (containing no interactive components or video).

##### No Intervention (Inactive Comparator)

A *no intervention* option will be used as an inactive comparator to the theory-driven intervention options and the generic risk alert. Randomizing to *no intervention* will control for the potential impact of being notified of heightened lapse risk, which could activate any pre-existing strategies to prevent lapse. When participants are randomized to the no-intervention inactive comparator, they will *not* receive any notification that the lapse risk is elevated.

### Web-Based BOT Used to Test the JITAI: Rx Weight Loss

Given that dietary lapses are specific instances of nonadherence to one or more BOT dietary goals, interventions examining and targeting lapses must be tested within the context of BOT so that participants have dietary goals to lapse from. Participants will be provided with a well-established web-based BOT called Rx Weight Loss (RxWL). The RxWL program was initially developed to facilitate weight loss in 154 primary care patients, whose mean weight loss was 5.8% (SD 4.4%) of the initial body weight at 3 months that was maintained for an additional 3 months [52]. Since then, RxWL has been refined and tested in multiple contexts (eg, worksites and community settings) and consistently produces similar weight losses [53-55].

The RxWL program begins with a 30-minute introductory session in which a member of the research team introduces the program eating and activity goals, teaches the participants how to use RxWL, and provides brief instructions on self-monitoring. Participants are given a goal of losing 1 to 2 pounds per week to achieve a total weight loss of approximately 5% to 10% of their initial body weight. In order to achieve weight loss, they are prescribed a calorie goal of 1200 to 1800 kilocalories per day tailored to their initial weight, given guidelines to follow a low-fat or Mediterranean diet [56-58] and asked to gradually increase their physical activity to 200 minutes per week of aerobic exercise [59]. Participants are asked to self-monitor their daily weight, daily dietary intake, and daily physical activity [60]. Following the introductory session, RxWL consists of 12 weekly 10- to 15-minute multimedia lessons for training in behavioral strategies for healthy eating and physical activity. Lessons are interactive to improve patient engagement; they incorporate video, animation, audio, quizzes, and exercises for goal-setting, planning, and problem-solving [61]. Topics are drawn from gold standard, empirically supported weight management programs such as Look AHEAD and Diabetes Prevention Program [62,63] and include meal planning, developing an exercise schedule, restaurant eating, changing the home environment, obtaining social support, and weight loss maintenance. Each week, participants submit daily values for tracked weight, caloric, and physical activity minutes to the RxWL platform (or this information can be automatically shared with RxWL if the participant chooses to use the Fitbit app for self-monitoring). Participants then receive automated feedback messages on their progress to date in the form of text appearing on the RxWL platform. Messages contain encouragement for meeting weight, diet, and activity goals, as well as strategies to improve weight loss if goals are not met. As dietary feedback is based on average weekly caloric intake, RxWL feedback is distinct from interventions provided within the JITAI for dietary lapses, which focus on lapses and triggers occurring at specific moments in time. To ensure adequate engagement with RxWL, email reminders will be sent to participants who have not visited the platform in a given week. Participants will use RxWL for 3 months and then be asked to continue to follow the dietary recommendations and self-monitoring during the 3-month follow-up period (during which time they will be receiving the JITAI with no access to RxWL).

### Stage 1: Technical Preparation and Refinement

Although many of the components of the JITAI for dietary lapses have been extensively piloted, this will be the first time that it is being hosted on the PiLR Health platform, which ultimately supports improved scalability and delivery in future work. The first phase of this trial will therefore consist of a small refinement study to ensure proper functionality of the JITAI in PiLR Health and to identify any barriers to implementing the study protocol (eg, microrandomization and assessment procedures). Participants from the target population (n=15) will complete the trial protocol procedures as described below for 3 months. Participants will complete study assessments, which include questionnaires and wearing a wrist-based device to passively sense eating behavior at baseline and at 3 months. Semistructured interviews will be used to collect feedback at the 3-month assessment visit, during which time participants will be queried to identify initial problems and potential solutions related to using the JITAI in conjunction with the web-based BOT. Problems that arise during stage 1 will be resolved before commencing stage 2 (the fully powered MRT).

### Stage 2: The Microrandomization Trial

#### Overview

Stage 2 will consist of an MRT to evaluate the effects of 4 theory-driven interventions, generic risk alerts, or no intervention on the immediate occurrence of dietary lapse during a 6-month web-based BOT. The participants (n=159) will receive 3 months of web-based BOT + JITAI, followed by 3 months of JITAI only. The follow-up period allows the JITAI to be evaluated during active BOT and JITAI-only follow-up (during which time participants may choose to pursue continued weight loss or weight loss maintenance). The MRT includes sequential randomization to intervention options each time the JITAI identifies heightened lapse risk. The participants will attend an in-person orientation session, followed by baseline, 3-month, and 6-month assessments. The primary proximal outcome is dietary lapse (assessed via EMA after the randomization of intervention options). The secondary proximal outcome is eating characteristics (measured via wristwatch device at assessments) following the randomization of intervention options. Contextual moderators, such as location, time of day, whether the participant is in active BOT or follow-up, and the type of lapse trigger will be collected to fulfill the exploratory aim of this project. Information regarding JITAI engagement and satisfaction, and weight change will be collected and used for descriptive purposes. To ensure the safety of participants and staff during the COVID-19 pandemic, procedures have been designed such that they can be conducted via remote means (eg, video calls for study appointments and wireless scales) and in person. Participants will be compensated for completing the study appointments, completing EMA surveys, and wearing the wristwatch device.

#### Participant Eligibility Criteria

The following eligibility criteria ensure a generalizable sample of individuals with CVD risk who are interested in and would benefit from weight loss. Inclusion criteria are as follows: BMI between 25 and 50 kg/m^2^; age between 18 and 70 years; physician-confirmed diagnosis of prediabetes, type 2 diabetes mellitus, hypercholesterolemia, or hypertension; able to walk 2 city blocks without stopping; and English language fluency and literacy at the 6th grade level. Exclusion criteria are as follows: currently participating in another weight loss program; currently taking weight loss medication; having lost >5% of body weight in the 6 months before enrollment; pregnant within the 6 months before enrollment; plans to become pregnant within 6 months of enrollment; endorses experiencing chest pain during periods of activity or rest, or loss of consciousness in the 12 months before enrollment; endorses any medical condition that would affect the safety of participating in unsupervised physical activity; history of bariatric surgery; and endorses any condition that would result in an inability to follow the study protocol, including terminal illness, substance abuse, eating disorder (not including binge eating disorder), and untreated major psychiatric illness.

#### Recruitment and Enrollment

Participants will be recruited via advertisements in local media (eg, newspapers and radio), targeted web-based advertising (including social media), flyers and advertisements posted in waiting rooms and examination rooms in primary care offices, referrals from physicians within the Lifespan health system and hospital network, informational materials made available as part of the health and wellness program for employees in the hospital network, and direct mailings. Recruitment of men and minorities will be maximized by tailoring the advertisement content and placement. Interested individuals will be given a brief study description and screened via a web-based survey or telephone to determine eligibility. Those who appear eligible will be invited to attend an orientation session, where the study will be described, informed consent will be obtained, and BMI will be confirmed via height and weight measurements. Before returning for the baseline visit in approximately 1 week, participants will be asked to have their physician sign a permission form that confirms their CVD diagnosis as well as safety to participate in the weight loss program, complete baseline questionnaires, adequately record dietary intake for 7 days (at least two meals or snacks per day), and complete 7 days of the JITAI EMA protocol (at least 70% of EMA surveys completed). These procedures ensure that only eligible participants who are capable and willing to adhere to study procedures move forward with the remainder of the study. At the baseline appointment, participants receive a 30-minute introductory session to the web-based BOT and training in using the JITAI for dietary lapses.

#### Microrandomization

Sequential randomization (or microrandomization) to intervention options will occur via an algorithm that was created by the research team and embedded within the PiLR Health system server. Microrandomization begins at the start of the third week after participants have completed 2 weeks of EMA without any interventions on their dietary lapses and relevant triggers. PiLR Health will then use the algorithm and accrued participant data to microrandomize the delivery of interventions at each decision point (ie, when a participant is determined to be at risk for lapse after completing an EMA survey). The randomization is independent of prior randomization and participants’ responses to previously delivered interventions for lapse [18]. On the basis of pilot work, the predictive algorithm that guides the decision rule in this JITAI is expected to predict a heightened state of lapse risk approximately 1 to 2 times per day *on average* across participants [15]. This estimated average accounts for potential decreases in EMA adherence and the likelihood of lapse that may occur during the study. As such, each participant will likely be randomized to an intervention option approximately 180 times over the study period. In accordance with the primary aim to compare the immediate, proximal effect of any active intervention option as compared with no intervention, intervention options will be randomized based on the following probabilities: 0.4 of decision points will be randomized to no intervention (inactive control), 0.12 to generic risk alerts (active control), 0.12 to the enhanced education intervention option, 0.12 to the self-efficacy intervention option, 0.12 to the autonomous motivation intervention option, and 0.12 to the self-regulation intervention option. As such, a given participant is expected to receive no intervention at approximately 72 decision points over the study, and the remaining 108 decision points will be divided equally among the 5 remaining intervention options (approximately 21-22 each).

### Measures

Participants will complete assessments with a research assistant who does not need to be blinded because of sequential randomization at baseline, 3 months, and 6 months to complete the measures. Outcomes collected via the JITAI EMA will occur 6 times per day over the 6-month study period.

#### Primary Proximal Outcome Measure

As in several previous trials conducted by the research team and others, dietary lapses will be assessed via EMA [6,7,64]. EMA typically captures naturalistic eating behavior better than lab-based tasks because near real-time reporting has the potential to reduce bias and improve validity [13,65,66]. Participants will be asked at each EMA survey to report whether they have experienced a dietary lapse since the last survey. A dietary lapse will be defined as any “eating or drinking likely to cause weight gain and/or put weight loss/maintenance at risk.” Participants will be asked to record the time of the lapse and will be asked to describe the lapse using the following select all-that-apply options: “I ate a larger portion of a meal or snack than I intended,” “I ate when I had not intended to eat,” or “I ate a type of food that I intended to avoid.” Participants will be trained to identify and report dietary lapses at the baseline visit and retraining will occur at 3- and 6-month visits.

#### Secondary Proximal Outcome Measures

Wrist-based accelerometry will be used to passively infer the frequency of eating, duration of eating episodes, rate of eating, and estimated count of bites during eating. The goal of including these objectively measured eating characteristics is to examine the potential effects of the JITAI intervention options on eating behaviors that are difficult to capture via self-report (eg, longer duration of eating [67], slower eating [68], more regular eating patterns [69], and more bites per meal). Participants will wear the ActiGraph GT9X Link (ActiGraph, LLC) on their dominant wrist for 2 weeks at each assessment point (first 14 days of treatment, 3 months, and 6 months). Although the ActiGraph is typically used to measure physical activity and sleep, the inertial measurement unit, which contains an accelerometer and a gyroscope, allows for the detection of a characteristic wrist-roll motion that occurs when food is brought to the mouth. These data will be analyzed using eating detection and characterization algorithms that have been extensively developed and validated by the research team [20,70-73]. These studies have shown that wrist-roll patterns and velocity can be analyzed to infer the timing and duration of eating with approximately 81% accuracy and estimate the number of bites taken during a meal with 86% sensitivity [20,71]. Both metrics can then be used to calculate the rate of eating (seconds/bite) [20]. After inferring and characterizing eating episodes, the following variables will be calculated: number of eating episodes, the average duration of eating episodes, total duration of eating, average bites taken during each episode, total bites taken, and the average rate of eating. During the baseline assessment period, these variables will be calculated at the day level and used descriptively because microrandomization will not take place. During the 3- and 6-month assessment periods, these variables will be calculated at the level of microrandomization (eg, 2-3 hours between intervention access and the next EMA survey).

#### Tailoring Variables

Each JITAI EMA prompt will measure tailoring variables that have been previously validated for lapse prediction across several pilot studies [7,15,23]. The data will be used by the predictive decision rule algorithm to determine whether an individual is likely to be in a state of heightened lapse risk. The following tailoring variables will be assessed: hunger, cravings, missed meals or snacks, presence of tempting food, urges to eat, socializing (with and without food), watching television, affect, negative interpersonal interactions, seeing advertisements for food, hours of sleep, fatigue, confidence, planning meals and snacks, boredom, cognitive load (ie, amount of cognitive difficulty during everyday tasks), level of motivation for weight loss, alcohol consumption, and time of day (automatically recorded by PiLR Health). Each EMA question and the respective response options that will be used to measure the tailoring variables are featured in Table 1. As described in the analytic plan, tailoring variables will also be evaluated as exploratory contextual moderators (eg, if the presence of a particular trigger impacts the efficacy of intervention options).

**Table 1:** Just-in-time adaptive intervention tailoring variables that inform the determination of heightened states of risk for dietary lapse.

Tailoring variable	Ecological momentary assessment question	Response options
Missed meal or snack	“Have you eaten since the last survey?”	Yes No
Affect	“Please rate your current mood”	I am in an especially good mood I am in a good mood I feel slightly stressed/upset I feel very stressed or upset I feel intensely stressed or upset
Fatigue	“Do you feel tired right now?”	Yes No
Hunger	“Are you hungry right now?”	Yes No
Boredom	“Are you bored right now?”	Yes No
Motivation for weight loss	“Compared with other things in your life, is weight control a high priority for you right now?”	Yes No
Cravings	“Are you experiencing a craving (an intense desire or urge to eat a specific food) right now?”	Yes No
Urges to eat	“Since the last survey, have you had a sudden urge to go off your eating plan for the day?”	Yes No
Cognitive load	“Since the last survey, please rate the difficulty of tasks that you have been working on in terms of the mental effort required (eg, work, planning, decision-making).”	Requiring almost no mental effort Requiring slight mental effort Requiring moderate mental effort Requiring most of my mental effort Requiring almost all of my mental effort
Confidence	“How confident are you that you can meet your dietary goals for the rest of the day?”	Not at all A little bit Somewhat A lot Very Extremely
Socializing	“Since the last survey, have you engaged in socializing with coworkers, family, or friends?”	None Yes, and there was food present Yes, and there was not food present
Watching television	“Since the last survey, have you watched TV?”	Yes No
Interpersonal interactions	“Since the last survey, have you had an unpleasant encounter with another person?”	Yes No
Presence of tempting foods	“In the past hour, would it have been easy to access delicious (but unhealthy) food/drink?”	Yes No
Food advertisements	“In the past hour, have you seen an advertisement for food?”	Yes No
Planning meals or snacks	“To what extend have you planned your eating in the next few hours?”	Not at all Slightly Moderately Very Extremely
Alcohol consumption	“Since the last survey, have you consumed any alcohol?”	Yes No
Sleep	“How many hours of sleep did you have last night?”	(Numeric response)
Time of day	Automatically recorded by the PiLR app	Automatically recorded in PiLR

#### Contextual Moderators

Contextual moderators will be used to further optimize intervention delivery within the JITAI. In addition to informing the JITAI decision rule, the above-described tailoring variables will be evaluated as contextual moderators (eg, if the presence of a particular trigger impacts the efficacy of intervention options). In addition, analyses will explore potential moderators of location (self-reported via EMA as described in Table 1) and whether the participant is in active BOT or JITAI-only follow-up.

#### Measures for Descriptive Purposes

##### Engagement and Satisfaction

Engagement with the JITAI (ie, the degree to which surveys and interventions within the JITAI were completed) will be assessed via PiLR Health. The following information will be automatically timestamped by the server: EMA surveys delivered, EMA surveys completed, interventions delivered, interventions accessed, and any responses recorded in interactive content. From this information, the percentage of EMA surveys completed, percentage of interventions accessed, and percentage of interventions with recorded participant interaction will be calculated. Participants will be asked to indicate satisfaction with the intervention content using a 5-star rating system (1 star is least helpful and 5 is the most helpful) at the conclusion of each module [74].

##### Participant Characteristics

Demographic information, health, and weight history will be assessed at baseline. Weight will be measured to the nearest 0.1 kg using a digital scale at each assessment; height will be measured to the nearest millimeter with a stadiometer at baseline, using standard procedures. Measurements will be made in light indoor clothing without shoes. Height and weight are measured solely for descriptive purposes and are to be used in reporting.

### Analytic Plan, Sample Size, and Power Estimates

#### Analytic Plan

Statistical analysis will follow good practices for the evaluation of RCTs as embodied in the Consolidated Standards of Reporting Trials statement [75]. Preliminary analyses will include descriptive statistics and exploratory graphing for all variables of interest that are measured at all assessment points. Initial exploratory data analysis will be used to identify outliers, such as measurement and recording errors, logical inconsistencies in data, and values extreme in the marginal distributions of the variables in question. Key baseline variables (eg, baseline BMI, age, and sex) will be considered for use as covariates in the proposed analyses. Missing data will be imputed using a multiple imputation approach and outcome models averaged across imputations to adhere to the intent-to-treat principle. A sensitivity analysis will explore the impact of various assumptions about missing data on study results, including assumptions that the outcome (lapses) is missing not at random, as participants may be more likely to skip surveys when they have lapsed.

Generalized multilevel models will be used to evaluate the study aims [76,77]. These types of models allow for increased statistical power, account for a hierarchical data structure (eg, observations nested within individuals within days), and include all participants regardless of whether there are missing data at particular time points [78]. First, a generalized multilevel model will be used to examine the effect of any intervention (4 theory-driven interventions and generic risk alert) compared with the *no intervention* condition on the occurrence of lapse (aim 1). Whether an intervention is provided at a decision point will be used to predict each participant’s probability of reporting a lapse in the following EMA survey. Next, an interaction between the intervention indicator variable and the week in which the intervention occurred will be added to test time trends in intervention effects [79]. Different distributions and link functions will be evaluated by comparing and assessing model assumptions and goodness-of-fit measures. Restricted maximum likelihood will be used to estimate the model parameters and to test the significance of random effects. Statistical significance will be accepted when *P*<.05 (2-tailed) and the estimated coefficient for the predictor (without accounting for the time trend) will represent the overall (average across all decision points) effect of delivering any intervention versus providing no intervention on the probability of lapse.

Second, similar methods will be used to build generalized multilevel models that examine the efficacy of the 4 individual theory-driven intervention options, compared with the generic risk alert, on the immediate occurrence of lapse (aim 2). In total, 4 intervention indicator variables will be used separately to represent whether each of the 4 theory-driven interventions was provided at a decision point, which allows for the comparison of the average effects of the theory-driven intervention options versus the generic risk alert on the probability of reporting a lapse in the next EMA survey. A comparison among the intervention options will be informed by estimated effect sizes.

Aim 1 and aim 2 analyses will be repeated to evaluate the immediate effects of intervention options on objectively measured eating characteristics at 3 and 6 months. Generalized multilevel models will be used to examine the effects of intervention indicators on the number of eating episodes detected via ActiGraph between the decision point and the next EMA survey, and the duration, rate of eating, and number of bites taken per eating episode recorded during that period. Intervention indicators will be allowed to interact with the day of assessment (eg, day 1 vs day 14 of wear time) and assessment period (eg, baseline vs 3 months) to account for potential time trends in intervention effects. In addition to other key demographic variables (eg, baseline BMI, age, and sex), ActiGraph wear time (hours per day that the device was worn) will be considered as a potential covariate.

Third, potential contextual moderators (ie, time of day, location, active treatment vs follow-up, and type of lapse trigger) will be added to the above-described generalized multilevel models to further inform JITAI optimization (exploratory aim). Moderators will be allowed to interact with the intervention indicators to determine whether these variables moderate the effect of the intervention on probability of reporting a lapse in the next EMA survey. Meaningful moderators will have interaction terms that are statistically significant at the *P*<.05 level. Statistically significant interactions will be interpreted by plotting simple regression lines for each level of categorical variables or for high and low values of continuous variables. Given the exploratory nature of this aim, analyses will not formally control for multiple comparisons, but claims about results will be made with appropriate caution.

All analyses will be conducted on the intent-to-treat sample (every instance of microrandomization and subsequent intervention delivery will be included in the final analysis), and several assumptions about the missing data mechanism will be evaluated. Sensitivity to these assumptions will be tested by collecting follow-up information on all participants (including dropouts), and loss-to-follow-up censoring will be employed. In total, 3 statistical approaches for handling missing data will be compared: a multiple imputation approach to impute missing outcomes, inverse probability weighting with propensity scores to produce unbiased estimates provided that data are missing at random, and pattern mixture models to allow for the possibility that data are not missing at random.

#### Sample Size and Power Estimates

The sample size requirements of this trial were based on analyses proposed to accomplish both aim 1 (ie, compare the effects of no intervention option and any intervention option) and aim 2 (ie, compare the effects of theory-driven intervention options and the active comparator to one another). Statistical power and sample size were calculated according to the established procedures for powering MRTs, as described by Liao et al [17], which enable robust treatment effect estimation using the centered and weighted least squares method [80]. On the basis of previous work, participants are expected to average 180 points of randomization during the trial with an assumed 100% availability (because a participant in an algorithm-determined heightened state of lapse risk will have just completed an EMA survey, indicating that they are near a smartphone and able to engage [15]).

The sample size calculation began with aim 2, given that the minimum clinically significant difference among active intervention options will likely be smaller than comparing no intervention with any intervention. Using available data from previous studies, a standardized effect size of 0.1 for aim 2 was calculated (which corresponds to a minimum clinically significant difference of reducing lapses by an average of 1 lapse per week, with an SE of 3.27). A reduction of 1 lapse per week is estimated to be associated with an *additional* 2.6% weight loss over 6 months for a single intervention option, which could substantially boost the overall proportion of participants achieving meaningful weight losses. The estimated number of decision points available in which any of the 2 single intervention options were delivered is 43 (180 total decision points × 0.24 probability of delivering either of 2 intervention options). Thus, the required sample size to detect any given contrast between intervention options in aim 2 at 80% power and 0.05 type 1 error rate is 106. Inflating this number by 50% to account for the binary nature of the proximal outcome brings the required sample size to 159. For aim 1, a larger standardized effect size of 0.153 was estimated that corresponds to reducing lapses by an average of 2 lapses per week with an SE of 3.27. With the projected sample size of 159, type 1 error rate of 0.05, 180 decision points at which either intervention or no intervention was provided, and 0.60 probability of providing any intervention option, there will be at least 90% power to detect the specified effect for aim 1.

### Stage 3: Application of MRT Results for Optimization

Stage 3 of this trial will involve using the results of the MRT to inform additional algorithms that will ultimately optimize intervention delivery within the JITAI for dietary lapses. Results from aims 1 and 2 will contribute to an understanding of the most effective intervention for preventing the immediate, proximal occurrence of dietary lapse, whereas results from the exploratory aim will inform which interventions are effective in a particular context (eg, if the autonomous motivation intervention option is effective in the afternoon vs other times of day). Together, these findings will be used to optimize the current JITAI decision rule (ie, deliver an intervention whenever any participant is in a heightened state of lapse risk) by training the intervention delivery algorithm to also consider *contexts* in which *certain types* of intervention options should be delivered. The resulting new decision rule algorithm, ideally using models that are minimally computationally intensive and easy to interpret (eg, regressions and decision trees), will be dynamic and personalized by considering baseline variables (eg, sex, age, race, ethnicity, and baseline BMI), specific trigger types (eg, feelings of hunger vs feelings of boredom), and context (eg, location and time of day). The finalized JITAI will no longer randomize intervention but administer the intervention option likely to have the greatest effect under the current risk conditions. Stage 3 ensures the development of an optimal JITAI for dietary lapses that is ready to be tested in a future RCT to evaluate the effects on distal clinical outcomes such as weight and CVD risk.

## Results

This study was funded by the National Heart Lung and Blood Institute (Multimedia Appendix 1). As of the date of submission of this manuscript, the trial is ongoing. Data collection for stage 1 began on April 19, 2021, and has been completed. Stage 2 recruitment is scheduled to begin by October 1, 2021. As this research involves no more than minimal risk, there will be no interim analysis, and data and safety monitoring will occur in accordance with guidelines by the National Institutes of Health and the Institutional Review Board of record. Ethical approval was granted by the Miriam Hospital Institutional Review Board.

## Discussion

### Anticipated Findings

Overweight and obesity remain major public health concerns [81,82]. BOT is a recommended first-line treatment for weight loss and has the potential to reduce the severity of CVD risk factors [1-4]. However, nonadherence to the prescribed diet in BOT (ie, dietary lapse) has been shown to prevent many individuals from achieving expected weight loss outcomes [5,6]. Although gold standard BOT protocols typically provide behavioral strategies that are intended to promote dietary adherence (eg, stimulus control and meal planning), these interventions do not appropriately account for the complex, momentary, and dynamic nature of the numerous potential triggers of dietary lapses in everyday life [12]. Instead, a smartphone-based JITAI for dietary lapses that assesses potential triggers for lapse via EMA and provides intervention during heightened states of lapse risk, is a scalable approach that has shown promise for improving dietary adherence in BOT [15,16]. This clinical optimization trial represents a critical next step in developing this JITAI for dietary lapses, aiming to optimize the approach by empirically tailoring the provision of an evidence-based intervention to the individual and the context.

This study will use a JITAI to compare which theory-driven interventions (vs no intervention or a generic alert to lapse risk) have an immediate, proximal impact on dietary lapses and other characteristics of eating behavior (eg, rate of eating and bite count). The results will establish, for the first time, whether the provision of in-the-moment intervention during heightened states of lapse risk has a direct effect on preventing lapse. These data will inform the optimization and refinement of the JITAI by revealing which types of interventions and in what contexts have the greatest impact on lapse [18,79]. Using this formative work to optimize the JITAI now ensures that the intervention is maximally effective, efficient, and directly targets the proximal outcome of interest (ie, dietary lapses) before conducting a future RCT to evaluate the efficacy of the JITAI for improving weight loss and reducing CVD risk [18].

An MRT design will be used to examine immediate, proximal effects of intervention options on lapse and thus provide the necessary data to optimize the JITAI. Rather than randomizing an individual only once to a single treatment, as is typical in an RCT, the MRT uses sequential randomization to repeatedly randomize individuals to intervention at specific instances based on their current state or context (in the current trial and heightened lapse risk) [17,19]. As each participant will be randomized approximately 180 times over the course of the study, the design requires fewer participants to achieve sufficient power to detect the proximal main effect of an intervention option. In contrast, using a traditional RCT to optimize this JITAI would require numerous participants across 6 intervention conditions *and* would not be able to directly compare intervention options within subjects. The MRT is therefore a major strength of this research, as it accelerates the translation of research to practice by answering several research questions within one study using fewer participants than a traditional RCT [83,84].

This project will be the first known implementation of an MRT protocol to optimize a JITAI on the proximal outcome of eating behavior. Across the field of health behavior change, MRTs remain novel; there are several MRTs in the process of optimizing JITAIs in the areas of physical activity [85,86], smoking cessation [87,88], stress management [88], mood [86], medication adherence [89], and substance use [90,91]. A recently completed MRT to increase self-monitoring in a commercial wellness app for lifestyle behaviors found that sending prompts with tailored suggestions (vs tailored feedback) significantly increased the odds of self-monitoring and that the frequency of engagement with the app moderated this effect (eg, as frequency of self-monitoring increased and sending prompts with suggestions reduced the odds of engagement) [92]. The results of this trial demonstrate the way in which MRTs provide crucial information about how context impacts intervention efficacy. One of the most influential MRTs was the microrandomized optimization trial of HeartSteps, which has provided a guiding framework for harnessing the MRT design for JITAI optimization [18]. The trial evaluated the efficacy of different types of suggestions to increase physical activity via the HeartSteps mobile app [79]. The results revealed that providing a walking suggestion (vs no suggestion) increased step count by an average of 24% and that suggestions to reduce sedentary time did not affect step count. The study also found that the efficacy of suggestions in HeartSteps was initially stronger and diminished over the course of the study. The HeartSteps trial has informed several methodological and practical guidelines for executing MRTs [17,19,93,94] and provided a rich data source to optimize future versions of HeartSteps via innovative algorithms that personalize the content and timing of activity suggestions [95-97].

A major strength of the proposed JITAI for dietary lapses is the use of previously validated machine learning algorithms to determine the heightened states of lapse risk. Machine learning has enormous possibility for informing precision medicine tools; the ability to make sense of vast amounts of individual data through dynamic algorithms enables highly sophisticated and nearly automatic patient feedback [98-100]. One aim of the proposed MRT is to develop *additional* algorithms that can dovetail with the current decision rule infrastructure, resulting in an even more precise and potent JITAI for lapses. For example, data from this MRT can support simulation studies to develop reinforcement learning algorithms that continuously adapt the provision of support to changing contexts between and within individuals [96]. There is also potential for these data to be used in a *warm start* fashion, which would involve using participant data from this MRT to boost effective learning more quickly in future versions of the JITAI for lapses [97]. Both of these examples demonstrate how products resulting from this MRT can optimize future versions of this JITAI for lapses, but also lead to important methodological discoveries in using personal health data to inform precision medicine.

In addition, systematic evaluation of the efficacy of theory-driven interventions will allow the findings from this MRT to advance the science of dietary lapse etiology and prevention specifically and nonadherence more generally. As dietary lapses are relatively understudied, it is not known which theory-driven approaches to behavior change will be most effective for preventing lapses during heightened lapse risk [101,102]. The MRT will provide important data about the role of each theory-driven intervention in preventing lapse and how these roles may change over time and across different contexts [18]. For example, if the motivation intervention option is effective in reducing dietary lapse and this effect is moderated by whether a participant is in active treatment or follow-up, this might indicate that motivation is an important momentary factor contributing to adherence, *especially during JITAI-only follow-up.* The results of this MRT will therefore contribute to the development of more dynamic theories of adherence or nonadherence behavior by directly comparing the immediate effects of multiple behavior change theories repeatedly over the course of a behavioral intervention [11].

### Limitations

This study also has several notable limitations. First, the JITAI for dietary lapse is currently solely reliant on EMA, which improves the rigor of self-report but also incurs a high level of participant burden. Although, previous work indicates that participants are willing to respond to EMA prompts 6 times per day, there is a high priority for this research to transition to passively sensed dietary lapses or relevant triggers [64,103]. Second, the selected theory-driven intervention options to be evaluated in this MRT are based on the best available, but nonetheless static, model of adherence behavior. Without a dynamic model of behavior to guide the selection of intervention options, there is a risk that the interventions within this JITAI do not fully appreciate the known complexity of dietary adherence behaviors [101]. This MRT is designed such that the results are expected to inform dynamic models of behavior for future studies. Third, the analytic plan does not adjust for multiple comparisons with regard to the exploratory analyses that will be used for JITAI optimization. The results of these analyses will be interpreted with caution, and a distinction will be made between findings from the stated primary aims and exploratory analyses to develop and refine future iterations of the JITAI. Finally, study procedures have been modified such that they can be delivered in-person *and* remotely in response to the COVID-19 pandemic. The assessment of primary and secondary study outcomes, involving EMA and wrist-based eating detection and characterization, will remain unaffected by these changes, but other descriptive measures (eg, height and weight) may be affected.

### Conclusions

This project targets dietary lapses, which are a major cause of poor outcomes during BOT. An MRT will be used to test 4 possible theory-driven intervention options within a JITAI that monitors risk and intervenes on lapses as needed. The primary proximal outcome is the occurrence of dietary lapse, as measured via EMA, between when the intervention was delivered and the next EMA prompt. Secondary proximal outcomes of interest are objectively assessed eating characteristics via wrist-worn device. Contextual moderators of intervention efficacy, such as location and time of day, will be explored. Data from the MRT will inform additional algorithms to personalize the timing of intervention delivery, thus optimizing the JITAI such that it has the greatest potential to show clear clinical impact in future RCTs and pragmatic trials.

## Appendix

Multimedia Appendix 1 National Institutes of Health study section reviews.

## Abbreviations

BOT: behavioral obesity treatment
CVD: cardiovascular disease
EMA: ecological momentary assessment
IMS: Information-Motivation-Strategy
JITAI: just-in-time adaptive intervention
MI: motivational interviewing
MRT: microrandomized trial
RCT: randomized controlled trial
RxWL: Rx weight loss

## References

[1] Poirier P, Giles TD, Bray GA, Hong Y, Stern JS, Pi-Sunyer FX, Eckel RH (2006). Obesity and cardiovascular disease: pathophysiology, evaluation, and effect of weight loss. Circulation.

[2] Wadden TA, Foster GD (2000). Behavioral treatment of obesity. Med Clin North Am.

[3] Magkos F, Fraterrigo G, Yoshino J, Luecking C, Kirbach K, Kelly S, de Las Fuentes L, He S, Okunade A, Patterson B, Klein S (2016). Effects of moderate and subsequent progressive weight loss on metabolic function and adipose tissue biology in humans with obesity. Cell Metab.

[4] Lowe M (2003). Self-regulation of energy intake in the prevention and treatment of obesity: is it feasible?. Obes Res.

[5] Alhassan S, Kim S, Bersamin A, King AC, Gardner CD (2008). Dietary adherence and weight loss success among overweight women: results from the A TO Z weight loss study. Int J Obes (Lond).

[6] Forman EM, Schumacher LM, Crosby R, Manasse SM, Goldstein SP, Butryn ML, Wyckoff EP, Thomas JG (2017). Ecological momentary assessment of dietary lapses across behavioral weight loss treatment: characteristics, predictors, and relationships with weight change. Ann Behav Med.

[7] Goldstein SP, Zhang F, Thomas JG, Butryn ML, Herbert JD, Forman EM (2018). Application of machine learning to predict dietary lapses during weight loss. J Diabetes Sci Technol.

[8] Grilo CM, Shiffman S, Wing RR (1993). Coping with dietary relapse crises and their aftermath. Addict Behav.

[9] McKee H, Ntoumanis N, Smith B (2013). Weight maintenance: self-regulatory factors underpinning success and failure. Psychol Health.

[10] McGuire M, Wing R, Klem M, Hill JO (1999). Behavioral strategies of individuals who have maintained long-term weight losses. Obes Res.

[11] Spruijt-Metz D, Nilsen W (2014). Dynamic models of behavior for just-in-time adaptive interventions. IEEE Pervasive Comput.

[12] Spruijt-Metz D, Wen CK, O'Reilly G, Li M, Lee S, Emken BA, Mitra U, Annavaram M, Ragusa G, Narayanan S (2015). Innovations in the use of interactive technology to support weight management. Curr Obes Rep.

[13] Shiffman S, Stone AA, Hufford MR (2008). Ecological momentary assessment. Annu Rev Clin Psychol.

[14] Goldstein SP, Evans BC, Flack D, Juarascio A, Manasse S, Zhang F, Forman EM (2017). Return of the JITAI: applying a just-in-time adaptive intervention framework to the development of m-health solutions for addictive behaviors. Int J Behav Med.

[15] Forman E, Goldstein S, Zhang F, Evans B, Manasse S, Butryn M, Juarascio AS, Abichandani P, Martin GJ, Foster GD (2019). OnTrack: development and feasibility of a smartphone app designed to predict and prevent dietary lapses. Transl Behav Med.

[16] Goldstein SP, Thomas JG, Foster GD, Turner-McGrievy G, Butryn ML, Herbert JD, Martin GJ, Forman EM (2020). Refining an algorithm-powered just-in-time adaptive weight control intervention: a randomized controlled trial evaluating model performance and behavioral outcomes. Health Informatics J.

[17] Liao P, Klasnja P, Tewari A, Murphy SA (2016). Sample size calculations for micro-randomized trials in mHealth. Stat Med.

[18] Klasnja P, Hekler EB, Shiffman S, Boruvka A, Almirall D, Tewari A, Murphy SA (2015). Microrandomized trials: an experimental design for developing just-in-time adaptive interventions. Health Psychol.

[19] Seewald NJ, Smith SN, Lee AJ, Klasnja P, Murphy SA (2019). Practical considerations for data collection and management in mobile health micro-randomized trials. Stat Biosci.

[20] Dong Y, Scisco J, Wilson M, Muth E, Hoover A (2014). Detecting periods of eating during free-living by tracking wrist motion. IEEE J Biomed Health Inform.

[21] Nahum-Shani I, Smith S, Spring B, Collins L, Witkiewitz K, Tewari A, Murphy SA (2018). Just-in-time adaptive interventions (JITAIs) in mobile health: key components and design principles for ongoing health behavior support. Ann Behav Med.

[22] Stone A, Shiffman S (1994). Ecological momentary assessment (EMA) in behavorial medicine. Ann Behav Med.

[23] Goldstein S (2018). Comparing effectiveness and user behaviors of two versions of a just-in-time adaptive weight loss smartphone app. Dissertation.

[24] Roefs A, Boh B, Spanakis G, Nederkoorn C, Lemmens LH, Jansen A (2019). Food craving in daily life: comparison of overweight and normal-weight participants with ecological momentary assessment. J Hum Nutr Diet.

[25] Carroll E, Czerwinski M, Roseway A, Kapoor A, Johns P, Rowan K (2013). Food and mood: just-in-time support for emotional eating. Proceedings of the Humaine Association Conference on Affective Computing and Intelligent Interaction.

[26] Gonul S, Namli T, Baskaya M, Sinaci A, Cosar A, Toroslu I (2018). Optimization of just-in-time adaptive interventions using reinforcement learning. Proceedings of the International Conference on Industrial, Engineering and Other Applications of Applied Intelligent Systems.

[27] DiMatteo MR, Haskard-Zolnierek KB, Martin LR (2012). Improving patient adherence: a three-factor model to guide practice. Health Psychol Rev.

[28] Fisher W, Fisher J, Harman J (2003). The information-motivation-behavioral skills model: a general social psychological approach to understanding and promoting health behavior. Social Psychological Foundations of Health and Illness.

[29] Linn AJ, van Weert JC, Smit EG, Perry K, van Dijk L (2013). 1+1=3? The systematic development of a theoretical and evidence-based tailored multimedia intervention to improve medication adherence. Patient Educ Couns.

[30] Mael F, Jex S (2015). Workplace Boredom: an integrative model of traditional and contemporary approaches. Group Organ Manag.

[31] Abraham C, Michie S (2008). A taxonomy of behavior change techniques used in interventions. Health Psychol.

[32] Mehrabian F, Farmanbar R, Mahdavi-Roshan M, Omidi S, Aghebati R (2018). The effect of nutrition education based on DASH diet on blood pressure and dietary adherence among patients with hypertension. Caspian J Health Res.

[33] Greiner B, Wheeler D, Croff J, Miller B (2019). Prior knowledge of the Mediterranean diet is associated with dietary adherence in cardiac patients. J Am Osteopath Assoc.

[34] Negarandeh R, Mahmoodi H, Noktehdan H, Heshmat R, Shakibazadeh E (2013). Teach back and pictorial image educational strategies on knowledge about diabetes and medication/dietary adherence among low health literate patients with type 2 diabetes. Prim Care Diabetes.

[35] Dinh TT, Bonner A, Clark R, Ramsbotham J, Hines S (2016). The effectiveness of the teach-back method on adherence and self-management in health education for people with chronic disease: a systematic review. JBI Database System Rev Implement Rep.

[36] Warziski MT, Sereika SM, Styn MA, Music E, Burke LE (2008). Changes in self-efficacy and dietary adherence: the impact on weight loss in the PREFER study. J Behav Med.

[37] Anderson-Bill ES, Winett RA, Wojcik JR, Winett SG (2011). Web-based guide to health: relationship of theoretical variables to change in physical activity, nutrition and weight at 16-months. J Med Internet Res.

[38] Burke LE, Ewing LJ, Ye L, Styn M, Zheng Y, Music E, Loar I, Mancino J, Imes CC, Hu L, Goode R, Sereika SM (2015). The SELF trial: a self-efficacy-based behavioral intervention trial for weight loss maintenance. Obesity (Silver Spring).

[39] Harrison JA, Mullen PD, Green LW (1992). A meta-analysis of studies of the Health Belief Model with adults. Health Educ Res.

[40] Horne R, Weinman J (1999). Patients' beliefs about prescribed medicines and their role in adherence to treatment in chronic physical illness. J Psychosomat Res.

[41] O'Keefe D, Jensen J (2007). The relative persuasiveness of gain-framed and loss-framed messages for encouraging disease prevention behaviors: a meta-analytic review. J Health Commun.

[42] DiClemente CC, Corno CM, Graydon MM, Wiprovnick AE, Knoblach DJ (2017). Motivational interviewing, enhancement, and brief interventions over the last decade: a review of reviews of efficacy and effectiveness. Psychol Addict Behav.

[43] Hayes S, Strosahl K, Wilson K (2011). Acceptance and Commitment Therapy: The Process and Practice of Mindful Change.

[44] VanWormer JJ, Boucher JL (2004). Motivational interviewing and diet modification: a review of the evidence. Diabetes Educ.

[45] Zhang C, Leeming E, Smith P, Chung P, Hagger MS, Hayes SC (2017). Acceptance and commitment therapy for health behavior change: a contextually-driven approach. Front Psychol.

[46] Elfhag K, Rossner S (2005). Who succeeds in maintaining weight loss? A conceptual review of factors associated with weight loss maintenance and weight regain. Obesity Reviews.

[47] Wing RR, Papandonatos G, Fava JL, Gorin AA, Phelan S, McCaffery J, Tate DF (2008). Maintaining large weight losses: the role of behavioral and psychological factors. J Consult Clin Psychol.

[48] Wing RR, Epstein LH, Nowalk MP, Lamparski DM (1986). Behavioral self-regulation in the treatment of patients with diabetes mellitus. Psychol Bull.

[49] Wing RR, Tate DF, Espeland MA, Lewis CE, LaRose JG, Gorin AA, Bahnson J, Perdue LH, Hatley KE, Ferguson E, Garcia KR, Lang W, Study of Novel Approaches to Weight Gain Prevention (SNAP) Research Group (2016). Innovative self-regulation strategies to reduce weight gain in young adults: the study of novel approaches to weight gain prevention (SNAP) randomized clinical trial. JAMA Intern Med.

[50] Wing RR, Tate DF, Gorin AA, Raynor HA, Fava JL (2006). A self-regulation program for maintenance of weight loss. N Engl J Med.

[51] Epstein LH (1990). Ten-year follow-up of behavioral, family-based treatment for obese children. J Am Med Assoc.

[52] Thomas JG, Leahey TM, Wing RR (2015). An automated internet behavioral weight-loss program by physician referral: a randomized controlled trial. Diabetes Care.

[53] Leahey TM, Thomas G, Fava JL, Subak LL, Schembri M, Krupel K, Kumar R, Weinberg B, Wing RR (2014). Adding evidence-based behavioral weight loss strategies to a statewide wellness campaign: a randomized clinical trial. Am J Public Health.

[54] Ross KM, Wing RR (2016). Implementation of an internet weight loss program in a worksite setting. J Obes.

[55] Thomas JG, Goldstein CM, Bond DS, Lillis J, Hekler EB, Emerson JA, Espel-Huynh HM, Goldstein SP, Dunsiger SI, Evans EW, Butryn ML, Huang J, Wing RR (2021). Evaluation of intervention components to maximize outcomes of behavioral obesity treatment delivered online: A factorial experiment following the multiphase optimization strategy framework. Contemp Clin Trials.

[56] Esposito K, Kastorini C, Panagiotakos DB, Giugliano D (2011). Mediterranean diet and weight loss: meta-analysis of randomized controlled trials. Metab Syndr Relat Disord.

[57] Nielsen L, Riddle M, King JW, Aklin WM, Chen W, Clark D, Collier E, Czajkowski S, Esposito L, Ferrer R, Green P, Hunter C, Kehl K, King R, Onken L, Simmons JM, Stoeckel L, Stoney C, Tully L, Weber W, NIH Science of Behavior Change Implementation Team (2018). The NIH Science of Behavior Change Program: transforming the science through a focus on mechanisms of change. Behav Res Ther.

[58] Shick SM, Wing RR, Klem ML, McGuire MT, Hill JO, Seagle H (1998). Persons successful at long-term weight loss and maintenance continue to consume a low-energy, low-fat diet. J Am Diet Assoc.

[59] Piercy KL, Troiano RP, Ballard RM, Carlson SA, Fulton JE, Galuska DA, George SM, Olson RD (2018). The physical activity guidelines for Americans. J Am Med Assoc.

[60] Burke LE, Wang J, Sevick MA (2011). Self-monitoring in weight loss: a systematic review of the literature. J Am Diet Assoc.

[61] Norman GJ, Zabinski MF, Adams MA, Rosenberg DE, Yaroch AL, Atienza AA (2007). A review of eHealth interventions for physical activity and dietary behavior change. Am J Prev Med.

[62] Wadden TA, West DS, Delahanty L, Jakicic J, Rejeski J, Williamson D, Berkowitz RI, Kelley DE, Tomchee C, Hill JO, Kumanyika S, Look AHEAD Research Group (2006). The Look AHEAD study: a description of the lifestyle intervention and the evidence supporting it. Obesity (Silver Spring).

[63] Diabetes Prevention Program (DPP) Research Group (2002). The Diabetes Prevention Program (DPP): description of lifestyle intervention. Diabetes Care.

[64] Goldstein SP, Hoover A, Evans EW, Thomas JG (2021). Combining ecological momentary assessment, wrist-based eating detection, and dietary assessment to characterize dietary lapse: A multi-method study protocol. Digit Health.

[65] Tomiyama AJ, Mann T, Comer L (2009). Triggers of eating in everyday life. Appetite.

[66] Grilo CM, Shiffman S, Wing RR (1989). Relapse crises and coping among dieters. J Consult Clin Psychol.

[67] Hamada Y, Kashima H, Hayashi N (2014). The number of chews and meal duration affect diet-induced thermogenesis and splanchnic circulation. Obesity (Silver Spring).

[68] Spiegel TA, Kaplan JM, Tomassini A, Stellar E (1993). Bite size, ingestion rate, and meal size in lean and obese women. Appetite.

[69] Forslund H, Lindroos A, Sjöström L, Lissner L (2002). Meal patterns and obesity in Swedish women-a simple instrument describing usual meal types, frequency and temporal distribution. Eur J Clin Nutr.

[70] Shen Y, Salley J, Muth E, Hoover A (2017). Assessing the acacuracy of a wrist motion tracking method for counting bites across demographic and food variables. IEEE J Biomed Health Inform.

[71] Dong Y, Hoover A, Scisco J, Muth E (2012). A new method for measuring meal intake in humans via automated wrist motion tracking. Appl Psychophysiol Biofeedback.

[72] Sharma S, Jasper P, Muth E, Hoover A (2020). The impact of walking and resting on wrist motion for automated detection of meals. ACM Trans Comput Healthc.

[73] Scisco JL, Muth ER, Dong Y, Hoover AW (2011). Slowing bite-rate reduces energy intake: an application of the bite counter device. J Am Diet Assoc.

[74] Perski O, Short C (2021). Acceptability of digital health interventions: embracing the complexity. Transl Behav Med.

[75] Altman DG, Schulz KF, Moher D, Egger M, Davidoff F, Elbourne D, Gøtzsche PC, Lang T, CONSORT group (Consolidated Standards of Reporting Trials) (2001). The revised CONSORT statement for reporting randomized trials: explanation and elaboration. Ann Intern Med.

[76] Singer J, Willett J, Willett J (2003). Applied Longitudinal Data Analysis: Modeling Change and Event Occurrence.

[77] Snidjers T, Bosker R (1999). Multilevel Analysis: An Introduction to Basic and Advanced Multilevel Modeling.

[78] Hayes AF (2006). A primer on multilevel modeling. Hum Comm Res.

[79] Klasnja P, Smith S, Seewald N, Lee A, Hall K, Luers B, Hekler EB, Murphy SA (2019). Efficacy of contextually tailored suggestions for physical activity: a micro-randomized optimization trial of HeartSteps. Ann Behav Med.

[80] Boruvka A, Almirall D, Witkiewitz K, Murphy SA (2018). Assessing time-varying causal effect moderation in mobile health. J Am Stat Assoc.

[81] Fryar C, Carroll M, Afful J (2020). Prevalence of overweight, obesity, and severe obesity among adults aged 20 and over: United States, 1960-1962 through 2017-2018. NCHS Health E-Stats.

[82] Cawley J, Biener A, Meyerhoefer C, Ding Y, Zvenyach T, Smolarz BG, Ramasamy A (2021). Direct medical costs of obesity in the United States and the most populous states. J Manag Care Spec Pharm.

[83] Collins Francis S, Riley William T (2016). NIH's transformative opportunities for the behavioral and social sciences. Sci Transl Med.

[84] Patrick K, Hekler E, Estrin D, Mohr D, Riper H, Crane D, Godino J, Riley WT (2016). The pace of technologic change: implications for digital health behavior intervention research. Am J Prev Med.

[85] Kramer J, Künzler F, Mishra V, Presset B, Kotz D, Smith S, Scholz U, Kowatsch T (2019). Investigating intervention components and exploring states of receptivity for a smartphone app to promote physical activity: protocol of a microrandomized trial. JMIR Res Protoc.

[86] Kroska EB, Hoel S, Victory A, Murphy SA, McInnis MG, Stowe ZN, Cochran A (2020). Optimizing an acceptance and commitment therapy microintervention via a mobile app with two cohorts: protocol for micro-randomized trials. JMIR Res Protoc.

[87] Dempsey W, Liao P, Kumar S, Murphy SA (2020). The stratified micro-randomized trial design: sample size considerations for testing nested causal effects of time-varying treatments. Ann Appl Stat.

[88] Battalio SL, Conroy DE, Dempsey W, Liao P, Menictas M, Murphy S, Nahum-Shani I, Qian T, Kumar S, Spring B (2021). Sense2Stop: a micro-randomized trial using wearable sensors to optimize a just-in-time-adaptive stress management intervention for smoking relapse prevention. Contemp Clin Trials.

[89] Li S, Psihogios AM, McKelvey ER, Ahmed A, Rabbi M, Murphy S (2020). Microrandomized trials for promoting engagement in mobile health data collection: adolescent/young adult oral chemotherapy adherence as an example. Curr Opin Syst Biol.

[90] Bell L, Garnett C, Qian T, Perski O, Potts HWW, Williamson E (2020). Notifications to Improve Engagement With an Alcohol Reduction App: Protocol for a Micro-Randomized Trial. JMIR Res Protoc.

[91] Rabbi M, Kotov MP, Cunningham R, Bonar EE, Nahum-Shani I, Klasnja P, Walton M, Murphy S (2018). Toward increasing engagement in substance use data collection: development of the substance abuse research assistant app and protocol for a microrandomized trial using adolescents and emerging adults. JMIR Res Protoc.

[92] Bidargaddi N, Pituch T, Maaieh H, Short C, Strecher V (2018). Predicting which type of push notification content motivates users to engage in a self-monitoring app. Prev Med Rep.

[93] Walton A, Nahum-Shani I, Crosby L, Klasnja P, Murphy S (2018). Optimizing digital integrated care via micro-randomized trials. Clin Pharmacol Ther.

[94] Qian T, Walton A, Collins L, Klasnja P, Lanza S, Nahum-Shani I, Rabbi M, Russell MA, Walton MA, Yoo H, Murphy SA (2020). The micro-randomized trial for developing digital interventions: experimental design and data analysis considerations. arXiv.

[95] Liao P, Dempsey W, Sarker H, Hossain SM, al'Absi M, Klasnja P, Murphy S (2018). Just-in-time but not too much. Proc ACM Interact Mob Wearable Ubiquitous Technol.

[96] Liao P, Greenewald K, Klasnja P, Murphy S (2020). Personalized HeartSteps: a reinforcement learning algorithm for optimizing physical activity. Proc ACM Interact Mob Wearable Ubiquitous Technol.

[97] Zhu F, Lia P (2017). Effective warm start for the online actor-critic reinforcement learning based mhealth intervention. arXiv.

[98] Yanovski SZ, Yanovski JA (2018). Toward precision approaches for the prevention and treatment of obesity. J Am Med Assoc.

[99] Krittanawong C, Zhang H, Wang Z, Aydar M, Kitai T (2017). Artificial intelligence in precision cardiovascular medicine. J Am Coll Cardiol.

[100] Margolis R, Derr L, Dunn M, Huerta M, Larkin J, Sheehan J, Guyer M, Green ED (2014). The National Institutes of Health's Big Data to Knowledge (BD2K) initiative: capitalizing on biomedical big data. J Am Med Inform Assoc.

[101] Riley WT, Rivera DE, Atienza AA, Nilsen W, Allison SM, Mermelstein R (2011). Health behavior models in the age of mobile interventions: are our theories up to the task?. Transl Behav Med.

[102] Brawley LR, Culos-Reed S (2000). Studying adherence to therapeutic regimens. Controll Clin Trials.

[103] Crochiere R (2020). Integrating sensor technology and machine learning to target dietary lapses. Master's Thesis.

