# Supplementary material for: Optimizing a Just-in-Time Adaptive Intervention to Improve Dietary Adherence in Behavioral Obesity Treatment: Protocol for a Microrandomized Trial
Source: JMIR Res Protoc. 2021 Dec 6;10(12):e33568. doi: 10.2196/33568 (PMC8691411; doi:10.2196/33568)
Supplement: Multimedia Appendix 1 [file resprot_v10i12e33568_app1.pdf]

SUMMARY STATEMENT

**PROGRAM CONTACT:**	**(Privileged Communication)**	***Release Date:***	**02/05/2020**
		Revised Date:	**02/06/2020**

Application Number: **1 R01 HL153543-01**

Principal Investigator

GOLDSTEIN, STEPHANIE PAIGE

Applicant Organization: MIRIAM HOSPITAL

Review Group:	**PRDP**<br>**Psychosocial Risk and Disease Prevention Study Section**		
Meeting Date:	**01/27/2020**	***RFA/PA:***	**PA18-722**
Council:	**MAY 2020**	***PCC:***	**HHCNP N**
Requested Start:	**07/01/2020**		
Project Title:	**Optimizing Just-in-Time Adaptive Intervention to Improve Dietary Adherence in Behavioral Obesity Treatment: A Micro-randomized Trial**		
SRG Action:	**Impact Score:17 Percentile:5**		
Next Steps:	**Visit https://grants.nih.gov/grants/next_steps.htm**		
**Human Subjects:**	**30-Human subjects involved - Certified, no SRG concerns**		
**Animal Subjects:**	**10-No live vertebrate animals involved for competing appl.**		
**Gender:**	**1A-Both genders, scientifically acceptable**		
**Minority:**	**1A-Minorities and non-minorities, scientifically acceptable**		
**Age:**	**3A-No children included, scientifically acceptable**		

ADMINISTRATIVE BUDGET NOTE: The budget shown is the requested budget and has not been adjusted to reflect any recommendations made by reviewers. If an award is planned, the costs will be calculated by Institute grants management staff based on the recommendations outlined below in the COMMITTEE BUDGET RECOMMENDATIONS section.
EARLY STAGE INVESTIGATOR
NEW INVESTIGATOR

1R01HL153543-01 Goldstein, Stephanie

COMMITTEE BUDGET RECOMMENDATIONS
EARLY STAGE INVESTIGATOR
NEW INVESTIGATOR
REVISED 2/6/2020

RESUME AND SUMMARY OF DISCUSSION: This well written application posits that nonadherence to dietary recommendations is the enemy of successful obesity treatment outcomes. The review panel was very enthusiastic about this very high impact work. Many strengths were noted: the outstanding scientific premise that innovation is needed to tackle lapsed dietary adherence in light of the fact that over 50 percent of failed obesity treatments are associated with lapses from the recommended diet; the exceptional and productive PI and impressive, well trained team of investigators further strengthened by an exceptional research environment; an outstandingly innovative smart phone based JITAI EMA research design and very significant intervention that sends support at the time of a dietary lapse; and an excellent approach including the elegant smart phone based micro-randomized 4 arm trial, inclusion of JITAI and EMA methods, strong theory driven conceptual basis, and work informed by the PI's previous work. In sum, the review panel judged this to be very important high impact work with a high likelihood to be able to tease out the different obstacles related to dietary lapses in obesity treatment.. A few minor weaknesses were discussed: concern that the intervention delivery depends on participants providing information on EMA prompts about their dietary lapse, and this work reduces a self- monitoring approach potentially limiting sustainability. In sum, this work offers very high impact to elucidate new methods to improve adherence to behavioral obesity treatment protocols and ultimately other adherence protocols.

DESCRIPTION (provided by applicant): Behavioral obesity treatment produces clinically significant weight loss and reduced disease risk/severity for many individuals with overweight/obesity and cardiovascular disease. Yet, about half of patients fall short of expected outcomes, which can be largely attributed to lapses from the recommended diet. Our work has shown that dietary lapses (specific instances of nonadherence to dietary goals) are frequent during weight loss attempts (~3-4 times per week), associated with poorer weight losses, and triggered by momentary changing states (e.g., changes in mood or availability of palatable food). Thus, there is a clear need for innovative solutions that can provide dynamic in-the-moment interventions to improve adherence to the prescribed diet in obesity treatment. Our research team was the first to develop a smartphone-based just-in-time adaptive intervention (JITAI) that includes: 1) daily ecological momentary assessment (EMA; repeated sampling via mobile device) of relevant behavioral, psychological, and environmental triggers for lapse; 2) a machine learning algorithm that uses information gathered via EMA to determine real-time lapse risk; & 3) delivery of brief intervention during high-risk moments. Our pilot work revealed that the JITAI was feasible, acceptable, and produced reductions in average lapse frequency. However, we have not yet shown a direct effect of the JITAI on eating behavior in the moment of heightened lapse risk and know little about the types of interventions that are most effective for reducing lapse. We therefore propose to extend our research via a micro- randomized trial (MRT), a methodology that involves random assignment to intervention (or control) at a specific decision point, i.e., when our algorithm predicts heightened risk for a lapse. The MRT will determine whether a specific intervention in a specific moment had its intended effect. We will therefore port our JITAI to a more scalable online platform and conduct a MRT to evaluate the effects of a generic lapse risk alert message and theory-driven just-intime interventions on dietary lapses. After refinement testing with n=15 to ensure proper technical functioning of our updated JITAI, adults with overweight/obesity (n=159) will participate in a

wellestablished 12-week online obesity treatment program + JITAI, with 12 weeks of JITAI-only follow-up. When an individual is at risk for lapsing s/he will be randomized to no intervention, a generic risk alert, or one of 4 theory-driven interventions with interactive skills training. The outcome of interest will be the occurrence (or lack thereof) of dietary lapse, as measured both subjectively (i.e., via EMA) and objectively (i.e., via wrist- based intake monitoring), in the hours following randomization. Results of the MRT will inform an optimized algorithm for intervention delivery that will drive the finalized JITAI. A future RCT will compare weight loss in obesity treatment with and without the optimized JITAI. This highly innovative approach will advance the science of adherence by supporting the development of sophisticated theoretical models of adherence behavior and informing JITAIs that target adherence to other health behaviors (e.g., medication, activity goals).

PUBLIC HEALTH RELEVANCE: This project targets dietary lapses (specific instances of nonadherence to dietary goals), a major cause of poor outcomes during behavioral obesity treatment, which is recommended as a first-line intervention for cardiovascular disease. We propose to conduct a micro-randomized trial to empirically optimize a just-in-time adaptive intervention that monitors risk and intervenes on lapses as needed. By evaluating the immediate, proximal effect of four theory-driven interventions on lapse behavior, the project will: (a) produce a scalable, finalized JITAI that has the greatest potential to show clear clinical impact in future RCTs and pragmatic trials; and (b) inform the development of more sophisticated theoretical models of adherence behavior more broadly.

CRITIQUE 1

Significance: 3
Investigator(s): 1
Innovation: 3
Approach: 4
Environment: 1

Overall Impact: The underlying premise of the study is that non-adherence to dietary recommendations challenges the effectiveness of behavioral obesity treatment, and that novel dynamic strategies are needed to address non-adherence. The investigators propose to use an innovative micro-randomized trial (MRT) to test the efficacy of delivering any intervention (theory-driven interventions or generic risk alert) compared to no intervention on the occurrence of dietary lapse. In Aim 2, the investigators will specifically compare the effects of four theory-driven interventions to generic risk alerts on the occurrence of lapse. In Aim 3, the team will integrate the data from the MRT and optimize the algorithm that will be used in a future RCT focusing on weight-loss as endpoint. Potential contextual moderators (e.g., time, location, active BOT/follow-up) will be considered in an exploratory aim. This is an interesting proposal with a clear focus on dietary lapses in the context of behavioral obesity treatment. The PI (Dr. Goldstein) is a productive junior investigator with expertise in use of technology in behavioral obesity treatment. The proposed study builds upon Dr. Goldtsein’s ongoing F32, and she assembled a stellar team of co-investigators with excellent track-record in conducting work of similar size and scope. Combining machine learning and MRT to study withinsubject response to interventions (vs. control) is innovative. The overall approach is sound and rigorous (e.g., measuring both self-reported lapses via ecological momentary assessment and objective eating using accelerometry). Minor weaknesses include the high internal complexity of the interventions delivered (too much combined and tested all at once), the reliance on fairly static and linear models of rational decision-making process (Health Belief Model and Theory of Planned Behavior), and questionable use of EMA survey as decision points.

GOLDSTEIN, S

1. Significance:

Strengths

- Innovative solutions to address adherence to treatment recommendations is highly significant in potentially increasing effectiveness.
- Using MRT to inform development of JITAI adds to the significance is providing dynamic/flexible tools to predict and prevent behavior in the real world.

Weaknesses

- (Minor) Unclear whether the paternalism of constantly monitoring lapse risk for participants can result in over-reliance on intervention messages and leave individuals ill-prepared for challenges in the "real-world" (i.e., outside of the behavioral obesity treatment bubble).
- Examining individual x contextual moderators would have strengthen the significance in informing the "what-works-for-whom in what situation/context".

2. Investigator(s):

Strengths

- Dr. Goldstein is currently a post-doctoral fellow (probably not for long) with expertise in clinical psychology and obesity. She trained with outstanding mentors and has been very productive.
- The proposed research is aligned with the PI expertise, allowing her to develop her unique and independent niche.
- The PI assembled an exceptional team of investigators with expertise in technology to assess eating behavior, just-in-time adaptive intervention, weight control interventions, microrandomized trial design, machine learning.
- The application provides evidence of sufficient biostatistics support. Drs. Zhang and Klasnja will provide statistical support and they have expertise with within-persons sequential randomization.

Weaknesses •

None

noted

3. Innovation:

Strengths

- Combining machine learning and micro-randomized trial (MRT) to study within-subject response to intervention (vs. control) is quite elegant and a powerful use of technology for functional behavior analysis/assessment. **Weaknesses**
- (Easily addressed) The reliance on Health Belief Model and Theory of Planned Behavior (and 3 of the 4 resulting interventions) is not especially innovative in the context of a dynamic JITAI. Both models assume a linear and rational decision-making process and are not very useful for behaviors with high automaticity.

4. Approach:

Strengths

- Supplementing self-reported lapse with objective measurement increase the scientific rigor of the data collected.
- The investigators were diligent in carefully documenting previous work supporting the scientific premise of the research and the expertise of the team in carrying the proposed activities.
- The application clearly describes the analytic plan and the team made judicious use of allotted sections.
- The proposed research builds upon previous and ongoing research resources, partnerships and infrastructure to address the rigor of previous research (or lack thereof) in conducting a rigorous RCT and testing carefully selected outcomes.
- The conceptual model (Figure 1) clearly outlines the implementation sequence of the JITAI components.
- The short duration of the online obesity treatment is acceptable because of the study overarching goal (i.e., optimizing JITAI specifically to interrupt lapses).
- Leveraging the PiLR Health platform is an efficient use of resources. PiLR HEALTH platform has been previously used and tested. Using PiLR further provides ongoing infrastructure and resources to maintain platform/system and minimizes delays.

Weaknesses

- The internal complexity of the 4 interventions (each intervention is packed with modules, steps, options and levels) will make it difficult to tease-out what works and what doesn't. The number of "and/or" possible permutations is a bit overwhelming.
- Using responses to EMA survey as decision points is questionable for a few reasons. The act of stopping and answering survey questions may create enough friction (cool-off period) to snapout of a mindless lapse (the study will be able to test this). More problematic is that alert overload may result in people ignoring prompts to complete a survey and/or the intervention delivery. One could also argue that lapses are more likely to occur in situations when people (deliberately or not) disengage from monitoring (i.e., put their phone down in this context). In other words, rather than the alert preventing the lapse, the alert may be ignored because of high-risk situations. Since inputs are needed to produce supervised machine learning, the nonrandom scattering may weaken the predictive value of the algorithm.
- (Minor) The exploratory aim is a bit generic in merely listing examples of moderators tested.
- It would have been useful to have more information on socioeconomic characteristics of the projected sample, especially since participants of modest means may inform the development of the intervention, and in turn benefit from increased access.
- (Easily addressed) Hypotheses would have provided directionality to the aims. I understand the exploratory empirical approach ("let's see what the data shows") but based on the investigators' expertise, I imagine they have some inkling of the relationships between the selected theorydriven interventions and lapse occurrence (otherwise why selecting these interventions).

5. Environment:

Strengths

- Miriam Hospital and Brown University are ideal research environments in terms of facilities, infrastructures, resources, partnerships and intellectual collaborations required to conduct the proposed work.
- These research environments will also ensure that the PI will receive sufficient support and mentoring.

Weaknesses

- Minor - Descriptions of Clemson University and the University of Michigan are rather thin. I understand that the investigators did not want to be overly inclusive of resources and facilities not in use in the research, but a little bit more details around resources and infrastructure available to the co-investigators would have been useful.
- Inconsequential typo: Some leftover text appears in the description of Drexel facilities and other resources (i.e., reference to Dr. Butryn)

Study Timeline:

Strengths

- The timeline is clearly described (which is rare!) and realistic based on the experience of the investigators and ongoing available infrastructure and resources.

Weaknesses

- None noted

Protections for Human Subjects:

Acceptable Risks and/or Adequate Protections

- Potential risks are clearly described, and strategies proposed to mitigate the risks are appropriate.

Data and Safety Monitoring Plan (Applicable for Clinical Trials Only):

Acceptable ○ Detailed DSMP is provided with clear role allocation / management plan.

Inclusion Plans:

- Sex/Gender: Distribution justified scientifically
- Race/Ethnicity: Distribution justified scientifically
- For NIH-Defined Phase III trials, Plans for valid design and analysis: Not applicable
- Inclusion/Exclusion Based on Age: Distribution justified scientifically
- The investigators plan to recruit men and women (capping enrollment of women to include more male participants). They also plan to enroll at least 30% from NIH-identified minority groups. The focus of the intervention is to address dietary lapses in adults participating in behavioral obesity treatment. Young children and adolescents (individuals under the age of 18) are excluded from this study because these groups have different needs than the obesity treatment provided in the study.

Vertebrate Animals:

Not Applicable (No Vertebrate Animals)

Biohazards:

Not Applicable (No Biohazards)

Resource Sharing Plans:

Acceptable

- Sharing plan is clearly described. The team will make data and documentation available using best practices.

Budget and Period of Support:

Recommended budget modifications or possible overlap identified:

- Not clear whether yearly travel support for conferences is fully justified for co-I Dr. Klasnja (data won't be available in early years). Travel to Rhode Island would make more sense.

CRITIQUE 2

Significance: 1
Investigator(s): 1
Innovation: 1
Approach: 1
Environment: 1

Overall Impact: The proposed new investigator R01 seeks to evaluate a smartphone based JITAI EMA dietary lapse prevention intervention in adults (N=159) using a micro-randomized trial design. Overall, this is a strong application with many notable strengths. The use of the MRT design to allow for investigating multiple theoretical and generic message approaches to limiting lapses and the use of an objective measure of eating characteristics are major strengths. The new investigator is well positioned to conduct the proposed study and is surrounded by a strong group of co-investigators. Based on these strengths and many others, the proposed study will likely have a significant scientific impact on the field.

1. Significance:

Strengths

- Focus on dietary non-adherence and the “in the moment” monitoring and delivery of an intervention at the time of occurrence is as an important issue to address in adult weight loss studies
- Use of mobile technology, coupled with risk of lapse monitoring and subsequent delivery (or not) of one or more theoretically driven interventions is compelling
- Use of the MRT to test multiple theories to determine the “best” approach to preventing/mitigating lapses is a major plus

Weaknesses

- None noted

2. Investigator(s):

Strengths

- New investigator has a strong background and experience to lead the proposed study
- Strong study team with necessary experience to assist with the successful completion of the proposed study

Weaknesses •

None

noted

3. Innovation:

Strengths

- The use of MRT to evaluate multiple theoretical approaches or the generic prevention message to addressing lapses in dietary behaviors

Weaknesses •

None

noted

4. Approach: Strengths

- Use of the MRT to evaluate multiple approaches to addressing dietary lapses
- Use of an objective device to measure eating occasions
- Measure of other characteristics associated with lapse
- The use of risk assessment to trigger intervention messages **Weaknesses** • None noted

5. Environment:

Strengths

- Strong environment to conduct the proposed study. **Weaknesses**
- None noted

Study Timeline:

Strengths

- Sufficiently detailed **Weaknesses**
- None noted

Protections for Human Subjects:

Acceptable Risks and/or Adequate Protections

Data and Safety Monitoring Plan (Applicable for Clinical Trials Only):

Acceptable

Inclusion Plans:

- Sex/Gender: Distribution justified scientifically
- Race/Ethnicity: Distribution justified scientifically
- For NIH-Defined Phase III trials, Plans for valid design and analysis: Not applicable •
  Inclusion/Exclusion Based on Age: Distribution justified scientifically

Vertebrate Animals:

Not Applicable (No Vertebrate Animals)

Biohazards:

Not Applicable (No Biohazards)

Resource Sharing Plans:

Acceptable

Budget and Period of Support:

Recommend as Requested

CRITIQUE 3

Significance: 1
Investigator(s): 2
Innovation: 1
Approach: 2
Environment: 1

Overall Impact: This R01 proposes a micro-randomized trial to test and refine a JITAI that focuses on reducing dietary lapses. The study's premise is well-grounded in a rigorous literature base. The approach has many strengths and few weaknesses. The project will advance a theory based JITAI that has so far proven technically feasible. The team has made a wise decision to focus on testing intervention components with a micro-randomized trial at this stage, rather than prematurely proposing an RCT of the entire intervention package. Overall, the project has potential for very high impact.

1. Significance:

Strengths

- Reducing lapses in behavioral obesity treatment is a vexing issue, and research is badly needed in this area

- Study would take an important step towards refining JITAIs for obesity treatment **Weaknesses**
- None

2. Investigator(s): Strengths

- PI is a productive, well-trained junior investigator
- Team includes an electrical/computer engineer, a health tech expert, an EMA expert, a behavioral weight loss expert, and a biostatistician

Weaknesses

- None

3. Innovation: Strengths

- The use of micro-randomized trial design is innovative
- Few teams have worked on developing JITAIs, and they remain a novel intervention strategy

Weaknesses

- None

4. Approach:

Strengths

- Preliminary data suggest that most aspects of the project is feasible, including extended wearing of wrist-worn sensors.
- Study includes a brief pilot test to troubleshoot technical issues with the JITAI.
- Strong recruitment and retention plan
- Trial uses a rigorous micro-randomized trial design comparing 4 theory-based interventions to no intervention and a generic intervention.

Weaknesses

- Given that EMA is used to both deliver the intervention and assess the primary outcome (dietary lapses), there is high potential for less adherent participants to have more missing data. (minor weakness)
- There is limited description of the wrist-worn sensor will be interpreted in relation to the JITAI and the various intervention components (minor weakness)

5. Environment:

Strengths

- Miriam Hospital provides a strong environment for this project **Weaknesses**
- None

Study Timeline:

Strengths

- All relevant milestones are accounted for **Weaknesses**
- None

Protections for Human Subjects:

Acceptable Risks and/or Adequate Protections

- Minimal risk study

Data and Safety Monitoring Plan (Applicable for Clinical Trials Only):

Acceptable

- Study will have an independent data safety officer

Inclusion Plans:

- Sex/Gender: Distribution justified scientifically
- Race/Ethnicity: Distribution justified scientifically
- For NIH-Defined Phase III trials, Plans for valid design and analysis: Scientifically acceptable
- Inclusion/Exclusion Based on Age: Distribution justified scientifically
- Women and men aged 18-70 years, all race/ethnicities

Vertebrate Animals:

Not Applicable (No Vertebrate Animals)

Biohazards:

Not Applicable (No Biohazards)

Resource Sharing Plans:

Not Applicable (No Relevant Resources)

Budget and Period of Support:

Recommend as Requested

THE FOLLOWING SECTIONS WERE PREPARED BY THE SCIENTIFIC REVIEW OFFICER TO SUMMARIZE THE OUTCOME OF DISCUSSIONS OF THE REVIEW COMMITTEE, OR REVIEWERS' WRITTEN CRITIQUES, ON THE FOLLOWING ISSUES:

PROTECTION OF HUMAN SUBJECTS: ACCEPTABLE

INCLUSION OF WOMEN PLAN: ACCEPTABLE

INCLUSION OF MINORITIES PLAN: ACCEPTABLE

INCLUSION ACROSS THE LIFESPAN PLAN: ACCEPTABLE

COMMITTEE BUDGET RECOMMENDATIONS: The review panel noted concerns with excessive travel to conferences.

REVISION NOTE: Updated roster attached

Footnotes for 1 R01 HL153543-01; PI Name: Goldstein, Stephanie Paige

NIH has modified its policy regarding the receipt of resubmissions (amended applications). See Guide Notice NOT-OD-14-074 at http://grants.nih.gov/grants/guide/notice-files/NOT-OD14-074.html. The impact/priority score is calculated after discussion of an application by averaging the overall scores (1-9) given by all voting reviewers on the committee and multiplying by 10. The criterion scores are submitted prior to the meeting by the individual reviewers assigned to an application, and are not discussed specifically at the review meeting or calculated into the overall impact score. Some applications also receive a percentile ranking. For details on the review process, see http://grants.nih.gov/grants/peer_review_process.htm#scoring
